# *In vitro* and *in silico* evaluation of the schistosomicidal activity of eugenol derivatives using biochemical, molecular, and morphological tools

**DOI:** 10.1590/1678-9199-JVATITD-2021-0108

**Published:** 2022-07-01

**Authors:** Isabella Maria Monteiro de Souza, Romulo Dias Novaes, Reggiani Vilela Gonçalves, Felipe Leonardo Bley Fialho, Diogo Teixeira Carvalho, Thiago Belarmino de Souza, Danielle Ferreira Dias, Stefânia Neiva Lavorato, Raquel Lopes Martins Souza, Marcos José Marques, Aline Pereira Castro

**Affiliations:** 1 Institute of Biomedical Sciences, Federal University of Alfenas (Unifal), Alfenas, MG, Brazil.; 2 Department of Animal Biology, Federal University of Viçosa, Viçosa, MG, Brazil.; 3 Institute of Chemistry, Federal University of Alfenas (Unifal), Alfenas, MG, Brazil.; 4 School of Pharmaceutical Sciences, Federal University of Alfenas (Unifal), Alfenas, MG, Brazil.; 5 School of Pharmacy, Federal University of Ouro Preto, Ouro Preto, MG, Brazil.; 6 Center of Biological Sciences and Health, Federal University of Western Bahia (Ufob), Barreiras, BA, Brazil.; 7 Department of Pharmacy, University Center of Lavras (Unilavras), Lavras, MG, Brazil.

**Keywords:** Schistosoma mansoni, Eugenol, Schistosomiasis, Mechanism of action

## Abstract

**Background:**

Eugenol shows both antibacterial and antiparasitic activities, suggesting that it might be evaluated as an option for the treatment of praziquantel-resistant schistosome.

**Methods:**

The *in vitro* activities of three eugenol derivatives (FB1, FB4 and FB9) on adult worms from *Schistosoma mansoni* were examined by fluorescence and scanning electron microscopy to analyze effects on the excretory system and integument damage, respectively. Biochemical tests with verapamil (a calcium channel antagonist) and ouabain (a Na^+^/K^+^-ATPase pump inhibitor) were used to characterize eugenol derivative interactions with calcium channels and the Na^+^/K^+^-ATPase, while *in silico* analysis identified potential Na^+^/K^+^-ATPase binding sites.

**Results:**

The compounds showed effective doses (ED_50_) of 0.324 mM (FB1), 0.167 mM (FB4), and 0.340 mM (FB9). In addition, FB4 (0.322 mM), which showed the lowest ED_50,_ ED_90_ and ED_100_ (p < 0.05), caused the most damage to the excretory system and integument, according to both fluorescence and scanning electron microscopy analysis. The death of adult worms was delayed by ouabain treatment plus FB1 (192 *versus* 72 hours) and FB9 (192 *versus* 168 hours), but the response to FB4 was the same in the presence or absence of ouabain. Besides, no changes were noted when all of the eugenol derivatives were combined with verapamil. Moreover, FB1 and FB9 inhibited Na^+^/K^+^-ATPase activity according to *in silico* analysis but FB4 did not show a time-dependent relationship and may act on targets other than the parasite Na+/K+-ATPase.

**Conclusion:**

Eugenol derivatives, mainly FB4 when compared to FB1 and FB9, seem to act more effectively on the integument of adult *S. mansoni* worms.

## Background

The term neglected diseases refers to a group of infections caused by a number of pathogens, including protozoa, viruses, bacteria, and helminths. These diseases most often affect impoverished populations that lack adequate sanitation and that live in close contact with infectious vectors and domestic animals. Unfortunately, neglected diseases historically have not been considered priorities for pharmaceutical companies, with the result that the available treatment options are obsolete, precarious, outdated, and even nonexistent in some cases [1]. Schistosomiasis is one neglected tropical disease that is directly associated with poverty and underdevelopment. In addition, it disables or kills millions of people and represent an important medical need that remains unmet. Current estimates indicate that more than 240 million people worldwide are affected by this disease and that more than 700 million people live in areas where it is endemic [1]. 

Praziquantel (PZQ) is the only medicine used to treat schistosomiasis, the ineffectiveness of this medication against the young forms of schistosome worm as it develops in the human host and the appearance of strains tolerant to PZQ have motivated the search for new active compounds against these helminths [2]. Plants are particularly attractive sources for these compounds, as herbal medicines have been used for centuries to treat human diseases and health disorders, and many plant products and secondary metabolites have known antiparasitic activities [3]. One of these metabolites is eugenol (4-allyl-2-methoxyphenol), a natural monoterpene component obtained from clove oil [4].

Eugenol shows several biological properties, including antioxidant, anti-inflammatory, analgesic, antipyretic, antibacterial, antifungal, and antitumor activities [5]. It is used in medicine as a local antiseptic and anesthetic and is recognized by the US Food and Drug Administration as a naturally occurring and safe antioxidant compound [6]. In addition to its biological activities, eugenol and related eugenol derivatives also show antiparasitic activities, such as antitripanosomal [7], antimalarial [8], and antileishmanial [9] effects, as well as therapeutic potential against *Trypanosoma. cruzi* [7], molluscicidal effects against schistosome vectors [10], activity against schistosomula of *S. mansoni* [11] and a complementary antischistosomal agent [12].

In the present study, three compounds derived from eugenol were assayed *in vitro* to determine their antischistosomal effects and the effective dose capable of killing 50% (ED_50_) of the adult worms of *S. mansoni*. The damage caused to the excretory system and to the integument of *S. mansoni* was also evaluated using resorufin and Hoechst 33258 probes, respectively, as well as examination by scanning electron microscopy (SEM). The effects of eugenol derivatives on Ca^2+^-channel and Na^+^/K^+^-ATPase activity were also analyzed by biochemical methods and comparison to verapamil and ouabain, respectively, as antagonists against adult *S. mansoni* worms. Finally, the effects of eugenol derivatives on the Na^+^/K^+^-ATPase enzyme activity were analyzed *in silico*.

## Methods

### Chemical compounds

**Figure 1.**
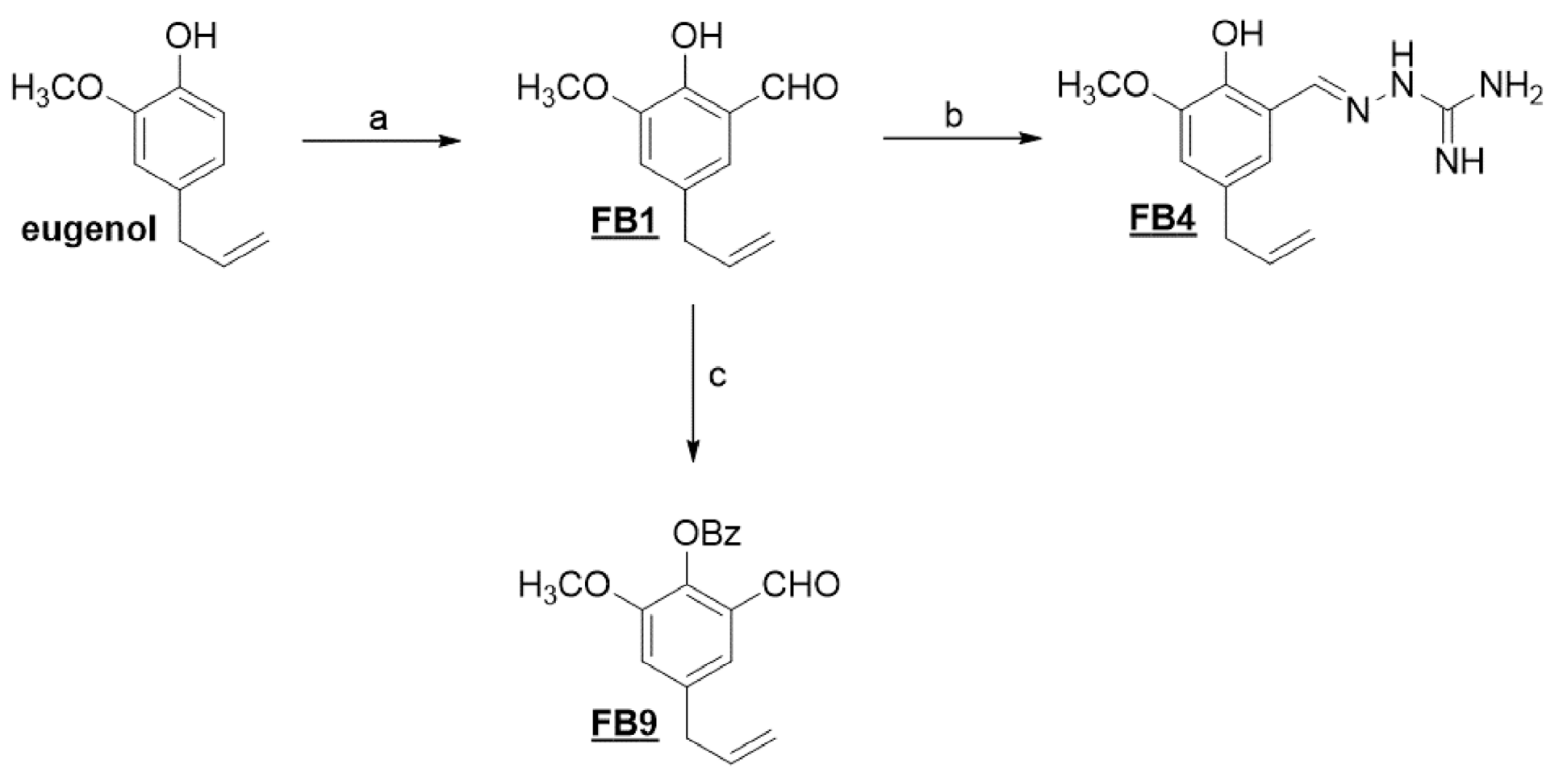
Reagents and conditions: **(a)** hexamine, acetic acid; **(b)** aminoguanidine bicarbonate, hydrochloric acid, ethanol; **(c)** benzoyl chloride, pyridine, dichloromethane.

Several eugenol compounds were synthesized for further biological analysis. The synthesis of eugenol derivatives (FB1, FB4, and FB9) is shown in Figure 1. A formyl eugenol derivative (FB1) was first obtained [13] and reacted with aminoguanidine bicarbonate [14] to form FB4, followed by acylation of the phenolic hydroxyl to yield a benzoyl derivative (FB9) [15]. All compounds were appropriately characterized.

### Synthesis and characterization

Reagents and solvents were purchased as reagent grade and were used without further purification. All derivatives were obtained from eugenol and were characterized by their nuclear magnetic resonance (NMR) and infrared (IR) spectra. 

*Synthesis of 5-allyl-2-hydroxy-3-methoxybenzaldehyde (FB1)*

Hexamethylenetetramine (60 mmol) and eugenol (12 mmol) were added to 10 mL of glacial acetic acid and stirred at 120°C for 5 h. After completion of the reaction (monitored by thin layer chromatography), the acetic acid was neutralized with a saturated sodium bicarbonate solution and this mixture was extracted with ethyl ether (4 × 50 mL). The organic phase was back-extracted with a 10% w/v sodium hydroxide solution (4 × 50 mL) and the aqueous phase was cooled and then acidified with concentrated hydrochloridric acid to pH 2. Subsequently, the acidified aqueous phase was extracted with dichloromethane (4 × 50 mL), and the organic phases were dried over anhydrous sodium sulphate, filtered, and evaporated under reduced pressure. The crude compound was purified by column chromatography (eluent hexane/ethyl acetate 7:3) to afford the desired product (light brown oil, 48% yield).

IR (ATR, cm^-1^): 3508 (O-H); 2937 (C-H); 2841 (C-H); 1651 (C=O); 1604 (C=C); 1463 (C=C); 1262 (C-O). ^1^H NMR (300 MHz, CDCl_3_) *δ*: 10.94 (1H, s, OH), 9.86 (1H, s, H-CHO), 6.97 (1H, dt, H-Ar, *J*= 2.1 and *J*= 0.6 Hz), 6.93 (1H, d, H-Ar, *J*= 2.1), 5.92-5.86 (1H, m, =CH), 5.12-5.06 (2H, m, =CH_2_), 3.89 (3H, s, OCH_3_), 3.36 (2H, d, CH_2_, *J*= 6,6). ^13^C NMR (75 MHz, CDCl_3_) *δ*: 196.6 (CHO), 150.04 (C-Ar), 148.2 (C-Ar), 136.72 (=CH); 131.42 (C-Ar), 123.7 (C-Ar), 120.45 (C-Ar), 118.7 (CH_2_), 116.52 (=CH_2_), 56.3 (OCH_3_), 39.4 (CH_2_).

*Synthesis of (E)-2-(5-allyl-2-hydroxy-3-methoxybenzylidene)hydrazine carboximidamide (FB4)*

Hydrochloridric acid (1 mol. L^-1^) was added dropwise to 10 mL of an ethanolic aminoguanidine bicarbonate (1.04 mmol) solution until gas evolution was complete. FB1 (0.52 mmol) was then added, and the solution was stirred for 12 h. The resulting precipitate was filtered and recrystallized in ethanol (white solid; 67% yield). 

IR (ATR, cm^-1^): 3429 (N-H); 3352 (O-H); 2962 (NH_2_); 1532 (C=N); 1363 (C=S); 1268 (C-O). ^1^H NMR (300MHz, DMSO-d_6_) *δ*: 3.27 (d, 2H, *J*= 2.1 Hz, CH_2_); 3.79 (s, 3H, OCH_3_); 4.99-5.10 (m, 2H, =CH_2_); 5.90-6.04 (m, 1H, =CH); 6.77 (d, 1H, *J*= 1.9 Hz, H-Ar); 7.36 (d, 1H, *J*= 1.9 Hz, H-Ar); 7.86 (s, 1H, NH_2_); 8.11 (s, 1H, NH_2_); 8.37(s, 1H, =CHN); 9.01 (s, 1H, NH); 11.37 (s, 1H, OH). ^13^C NMR (75MHz, DMSO-d_6_) *δ*: 39.9 (CH_2_); 55.9 (OCH_3_); 113.1 (C-Ar); 115.4 (=CH_2_); 117.6 (C-Ar); 120.4 (C-Ar); 130.4 (C-Ar); 138.1 (=CH); 139.6 (C-Ar); 144.3 (C-Ar); 147.9 (=CHN); 177.6 (C=NH).

*Synthesis of 4-allyl-2-formyl-6-methoxyphenyl benzoate (FB9)*

Benzoyl chloride (2.28 mmol) and 4-(dimethylamino) pyridine (0.23 mmol) were added to a solution of FB1 (1.9 mmol) in dichloromethane (25 mL). The reaction was stirred at room temperature for 3 h and monitored by thin layer chromatography. After completion of the reaction, ice cold water was added and the organic phase was washed with 0.5 mol/L sodium hydroxide solution (6 × 10 mL), followed by water until the pH reached pH 7. The organic phase was then dried over anhydrous sodium sulphate, and the solvent was removed under reduced pressure. The product was recrystallized in ethanol (white solid, 60% yield).

^1^H NMR (300MHz, DMSO-d_6_) *δ*: 3.49 (d, 2H, *J*= 6.9 Hz, CH_2_); 3.87 (s, 3H, OCH_3_); 5.17-5.15 (m, 1H, =CH_2_); 5.22-5.19 (m, 1H, =CH_2_); 6.07-5.94 (m, 1H, =CH); 7.11 (d, 1H, *J*= 2.1 Hz, H-Ar); 7.38 (d, 1H, *J*= 2.1 Hz, H-Ar); 7.59 - 7.54 (m, 2H, H-Ar); 7.70 (ttt, 1H, *J*= 1.6 and *J*= 7.5, H-Ar); 8.29 - 8.25 (m, 2H, H-Ar); 10.21 (s, 1H, CHO). ^13^C NMR (75MHz, DMSO-d_6_) *δ*: 40.2 (CH_2_); 56.6 (OCH_3_); 117.3 (=CH_2_); 118.6 (C-Ar); 120.4 (C-Ar); 128.9 (C-Ar); 129.4 (C-Ar); 130.7 (C-Ar); 134.2 (=CH); 136.5 (C-Ar); 139.5 (C-Ar); 140.9 (C-Ar); 152.0 (C-Ar); 164.9 (C-Ar); 188.9 (CHO).

### *In vitro* evaluation of eugenol derivatives

The *S. mansoni* LE (Luiz Evangelista) strain has been routinely maintained by serial passages in *Biomphalaria glabrata* models and in golden hamsters (*Mesocricetus auratus*) for more than 40 years in the Schistosomiasis Research group at the René Rachou Institute/Fiocruz Minas. The Research Ethics Committee from the Federal University of Alfenas (UNIFAL-MG) authorized all procedures under the registration number 25/2019 in accordance with the ethical principles required for animal research.

Mice infected with *S. mansoni* cercariae (LE strain) were euthanized 45 days after infection by an overdose of 100.0 mg/kg of ketamina, (Ketamina Agener^®^) and 10.0 mg/kg de xylazine hydrochloride (Rompun^®^) dissolved in saline solution (0.9% sodium chloride) and administered intraperitoneally. Subsequently, a retrograde liver perfusion was completed to obtain the adult worms [16]. The recovered parasites were cultivated in 24-well culture plates (one couple per well) in RPMI-1640 culture medium at pH 7.4 supplemented with 5.0% heat-inactivated fetal bovine serum (Sigma^®^, St. Louis, MO, USA), 1.0% penicillin (10,000 IU/mL), and streptomycin (10.0 mg/mL) (Sigma^®^, USA) for a thirty-minute adaptation period. A stock solution (4 mg/mL) with the eugenol derivatives (FB1, FB4 and FB9) was prepared using methanol as a solvent and were then added to the cultures at different concentrations for screening (150, 100, 75, and 50 μg/mL). The plates were kept in an incubator at 37°C and 5.0% CO_2_ and analyzed within 2 and 24 h after addition of the eugenol derivatives. The test groups were compared with the following control groups: RPMI-1640 supplemented medium, RPMI-1640 medium added with 25.0 μL of methanol (the vehicle used as solvent for the samples) and with PZQ (2 μg/mL or 0.0064 mM) under the same culture conditions. After 24 h, the wells were washed five times by removing the culture medium from the wells and adding the same amount of sterile medium to remove all traces of the added compounds. The cultures were analyzed daily for eight days (e.g., until 192h) with an inverted microscope (Nikon^®^ Eclipse TS100 microscope, magnification 4×, 10×, 20×, and 40×), and records of the adult worms were documented.

### Determination of effective dose (ED_50_)

The effective doses required to kill 50% (ED_50_) of the worms were determined using GraphPadPrism (version 5.0). The parasites were evaluated and compared with the controls in terms of the number of mated worms (percentage of paired couples), movement, contraction/shortening, morphology, shedding of the integument, and oviposition (percentage of presence *versus* absence), which, together were used to define the mortality. All tests were performed in triplicate as previous described (section “*In vitro* evaluation of eugenol derivatives”), with a couple of worms in each well totaling six worms in each experiment, and the experiments were carried out at three different times totaling eighteen worms; besides PZQ (at 0.0064 mM) was used as a pharmacological control drug. The ED_50_, ED_90_, and ED_100_ values were determined for the three eugenol derivatives using different concentrations (0.067 to 0.729 mM).

### Use of fluorescent probes for *in vitro* evaluation of the effects of eugenol derivatives on the excretory activity and integument damage in adult *S. mansoni* worms

The mechanism of action of eugenol derivatives was explored by evaluating the effects on the excretory system and the integument of the parasite using the fluorescent probes resorufin and Hoechst 33258, respectively. The parasites were marked with one of these fluorescent probes and then the effects of eugenol derivatives were evaluated by fluorescence microscopy. Resorufin is a fluorescent sodium salt (7-hydroxy 3-phenoxazine) that can act as a modulator/substrate for P-glycoprotein (Pgp), as described by Sato et al. [17]. Hoechst 33258 (bis-benzamide) (2,4-hydroxyphenyl-5,4-methyl-1-piperazine-2,5-bis-H-benzimidazole) is a hydrophilic probe and fluoresces only when it binds to the DNA of cells [18].

***Use of the resorufin probe to evaluate the activity of the excretory system of adult* S. mansoni *worms exposed to eugenol derivatives***

Adult worm couples were obtained and plated as previous described (section “*In vitro* evaluation of eugenol derivatives”). A 10.0 μL volume of resorufin (10 mg/mL stock solution) was added to wells containing two adult worms (one male and one female), the plates were incubated at 37°C and 5.0% CO_2_ for 30 min, and then 25.0 μL/mL of methanol, PZQ (0.0064 mM), FB1, FB4, or FB9 (at their respective ED_100_ concentrations) were added; control wells contained only culture medium. The plates were again incubated for 15 min, the parasites were washed 5 times with culture medium, and then slides were prepared for viewing with the fluorescence microscope. All tests were performed in triplicate (with a couple of worms in each well totaling six worms in each experiment, and the experiments were carried out at three different times totaling eighteen worms).

***Use of Hoechst 33258 staining to evaluate integument damage caused by exposure of adult* S. mansoni *worms to eugenol derivatives***

Adult worm couples were obtained and plated as previous described (section “*In vitro* evaluation of eugenol derivatives”). The plates were incubated for 24 h at 37º C with 5.0% CO_2_, the wells were washed as previously described, and then 10.0 μL of the Hoechst 33258 probe was added to each well and incubated for 15 min. The wells were washed again, and slides were prepared for viewing with the fluorescence microscope. All tests were performed in triplicate.

### Fluorescence microscopy

At the end of each experiment, the parasites were transferred to slides bounded by small amounts of petroleum jelly to prevent the parasites from spilling out from the slide. The parasites were placed on the slides with a small amount of culture medium and then were observed with an optical fluorescence microscope (Zeiss Axio^®^ Scope A1, Axio Vision LE software) using a rhodamine filter for resorufin (excitation/maximum emission of resorufin 571/585 nm) and DAP for Hoechst 33258 (Hoechst 352/455 nm maximum excitation/emission) to evaluate the damage caused to the excretory system and to the integument of *S. mansoni*, respectively, as described by Castro et al. [19].

### Scanning electron microscopy evaluation of eugenol derivative-induced integumentary damage

The morphological integrity of the integument of adult *S. mansoni* worms was evaluated by scanning electron microscopy (SEM) [20]. After the toxicity test, the worms were collected and fixed for 24 h in 10% buffered formalin (pH 7.2). The worms were dehydrated in a series of increasing concentrations of ethanol (1x at 50%, 70%, 90%, 95%, and 3x at 99.98%, 1h immersion in each concentration.) and dried in an oven at 60 ºC for 12 h. They were mounted on metallic supports, coated with gold (Modular Balzers Union FDU 010, SCA 010, Oerlikon Balzers, Balzers, Liechtenstein), and examined with a scanning electron microscope (Leo 1430VP; Carl Zeiss, Jena, Thuringia, Germany) [20]. The integumentary integrity was analyzed in worms treated FB1, FB4 and FB9 at four different concentrations, two below (FB1, 0.104 and 0.208 mM; FB4, 0.080 and 0.161 mM; FB9, 0.203 and 0.270 mM) and two above (FB1, 0.416 and 0.521 mM; FB4, 0.242 and 0.322 mM; FB9, 0.405 and 0.473 mM) the ED_50_. Toxic effects were defined from morphological evidence of integumentary erosion, peeling, bubbles, eruption, and contraction bands, as well as changes in the surface tubercle structure (collapse, fusion, and presence and distribution of spicules) [20,21].

### Comparison of the *in vitro* schistosomicidal activity of eugenol derivatives in the presence or absence of verapamil against adult *S. mansoni* worms

Adult worm couples were obtained and plated as previous described (section “*In vitro* evaluation of eugenol derivatives”). A 1.0 μL volume of verapamil (0.01 mM), used as calcium channel antagonist, was added to wells containing an adult worm couple and the plates were incubated in an oven at 37°C and 5.0% CO_2_ for 30 min. After this period, 6.5 μL of methanol, PZQ (0.0064 mM), or eugenol derivatives with their respective ED_50_ values (FB1, 0.324 mM; FB4, 0.167 mM; FB9, 0.340 mM) were added to the wells; control wells contained only culture medium, while the pharmacological control contained verapamil. The plates were incubated for a further 2 h, after that the plates were washed to remove the test substances and then the parasites viewed with an inverted microscope for assessment of motility and damage. The behavior of the adult worms was then assessed immediately and again 24, 48, and 72 h and 7 days later by observing the following parameters: i) mating; ii) movement; iii) shrinkage/shortening; iv) morphology; v) detachment of the integument; and vi) presence of eggs. The motility was evaluated using a semiquantitative scale, where 0 represents no activity; 1 is low; 2 is medium and 3 is intense motility.

### Comparison of the *in vitro* schistosomicidal activity of eugenol derivatives in the presence or absence of ouabain against adult *S. mansoni* worms

Adult worm couples were obtained and plated as previous described (section “*In vitro* evaluation of eugenol derivatives”). Ouabain (0.0063 mM) was used to block the Na^+^/K^+^-ATPase. The plates were incubated at 37°C and 5.0% CO_2_ for 30 min and then 40.0 μL of methanol (vehicle control), PZQ (0.0064 mM, pharmacological control), or ED_100_ concentrations of eugenol derivatives (FB1, 0.481 mM; FB4, 0.267 mM; FB9, 0.549 mM) were added; control wells contained only culture medium. The plates were incubated for a further 2 h and then the parasites were viewed with an inverted microscope to analyze motility and other damage. The motility was evaluated as described before. The test solutions were removed by washing, and the behavior of the adult worms was evaluated immediately and again 24, 48, and 72 h and 7 days later.

### *In silico* analysis

The structure of the alpha subunit of *S. mansoni* Na^+^/K^+^-ATPase was determined by homology modeling using the online server SWISSMODEL (https://swissmodel.expasy.org/). The model with the best parameters was refined using the GalaxyRefine online server (http://galaxy.seoklab.org/cgi-bin/submit.cgi?type=REFINE). A 1007 amino acid sequence was obtained from the UniProt database, code Q95WT4. A *Squalus acanthias* Na^+^/K^+^-ATPase alpha subunit (PDB ID 3A3Y) [22] was used as a template for homology modeling, as the subunit under study had a 74.32% sequence identity. The homology model was refined, and a Ramachandran graph was obtained, indicating 99% amino acids in favorable regions and 100% amino acids in allowed regions.

The structures of the three eugenol derivatives (FB1, FB4, and FB9) were drawn and optimized using BIOVIA Discovery Studio v19.1.0.18287. For optimization, a Dreiding-like force field was used. The homology model of the *S. mansoni* Na^+^/K^+^-ATPase alpha subunit was used for subsequent ligand-target interaction studies. The AutoDockTools 1.5.7 program [23] was employed to prepare PDBQT files of the macromolecule (addition of Gasteiger charges and atomic type AD4) and ligands (definition of torsional aspects). Docking studies were performed using AutoDock Vina 1.1.1 [24] with docking grid dimensions of 16 Å × 16 Å × 16 Å and coordinates of the center point at 146.941 × 15.247 × - 2.025, which covered the corresponding area of interaction of ouabain in the reference model (PDB ID 3A3Y). The ligand conformation that generated the enzyme-ligand complex with the lowest energy was used for analysis.

### Statistical analysis

Statistical analyzes were performed with GraphPad Prism (version 5.0). Linear regression was used to obtain the value of ED_50,_ ED_90_ and ED_100_. Significant differences were determined by means of one-way analysis of variance (ANOVA) followed by Tukey's test of multiple comparisons with a significance level of p < 0.05.

## 3. Results

### *In vitro* evaluation of eugenol derivatives

The ED_50_, ED_90_, and ED_100_ were determined for the eugenol derivatives against adult worms of *S. mansoni* as showed in the Table 1. The FB4 showed the lowest values to ED_50_, ED_90_, and ED_100_ in relation FB1 and FB9 (p < 0.05).

**Figure 2.**
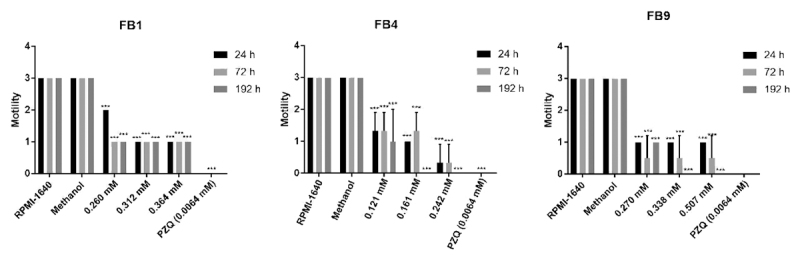
Motility of adult *Schistosoma mansoni* worms after exposure to different concentrations (0.260 to 0.364 mM for FB1; 0.121 to 0.242 mM for FB4 and 0.270 to 0.507 mM for FB9) of the eugenol derivatives for 24, 72 and 192 hours of incubation. RPMI-1640 and methanol were used as control and praziquantel (PZQ) as pharmacological control. ***Statistic analyses with significant result (p < 0.05) in relation to control groups.

The FB1 derivative caused a greater inhibition of the motility of adult worms in relation to RPMI-1640 and methanol groups at concentrations above 0.312 mM, with movement scores of 1 (low activity) in the first 24 h of incubation (Figure 2). On its turn, FB4 caused a reduction in motor activity at a concentration of 0.121 mM, with a score of 1 in the first 24 h of incubation and with concentrations above 0.161 mM it was possible to obtain the score 0 after 192 h (Figure 2). FB9 only showed a reduction in motor activity at concentrations above 0.270 mM and it was possible inhibit the motility of adult worms after 192 h at concentrations above 0.338 mM (Figure 2). The adult worms from the control groups (culture medium only or methanol) showed intense motility until the end of the observations (192 h) (Figure 2).

**Table 1.** ED_50_, ED_90_, and ED_100_ determined for the eugenol derivatives against adult worms of *S. mansoni* after 192 hours of contact with FB1, FB4, and FB9.

Compound	ED (mM)		
	50 (SD)	90 (SD)	100 (SD)
**FB1**	0.324 (± 0.025)^a^	0.449 (± 0.05)^a^	0.481 (± 0.056)^a^
**FB4**	0.167 (± 0.001)^b^	0.246 (± 0.007)^b^	0.267 (± 0.008)^b^
**FB9**	0.340 (± 0.011)^a^	0.515 (± 0.017)^a^	0.549 (± 0.018)^a^

ED: effective dose; SD: standard deviation; ^a,b^: statistical analyses with significant results (p < 0.05).

The effects of the eugenol derivatives were also investigated on the parameters of mating rate and egg laying from *S. mansoni* females (Table 2). Overall, 75.0, 75.0 and 50.0% of the worm couples exposed (at ED_50_ concentration) to FB1, FB4 and FB9, respectively, were separated up the last day of readings (192 h of incubation). Besides, no oviposition was observed during the 192 h even in the situation of mating (Table 2). The PZQ treatment caused static paralysis of the adult worms, followed by death. The PZQ-treated worms mated but laid no eggs (Table 2 **).** The adult worms in the control groups (culture medium only or methanol only) showed intense motility, active excretory systems, mating, and ovipositing, and had undamaged integument.

**Figure 3.**
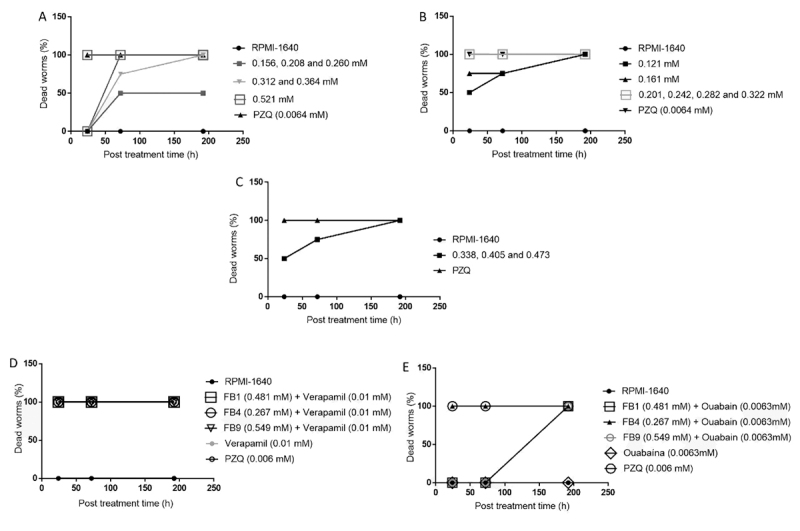
Effect of **(A)** FB1, **(B)** FB4, and **(C)** FB9 on the mortality rate of *Schistosoma mansoni* adult worms in relation to the assessed concentrations (0.156 to 0.521 mM for FB1; 0.121 to 0.322 mM for FB4, and 0.338 to 0.473 mM for FB9) and incubation periods of up to 192 hours. Pharmacological controls were exposed to 0.0064 mM PZQ. Effect of eugenol derivatives on the mortality rate of adult *S. mansoni* worms in contact with **(D)** verapamil and **(E)** ouabain.

The most significant results in relation to RPMI-1640 group (p < 0.05) were observed following exposure of the parasites to FB4 at concentrations of 0.201, 0.242, 0.282, and 0.322 mM where 100% mortality of male and female worms was observed in the first hours of incubation (Figure 3). A concentration of FB1 of 0.521 mM also caused 100% mortality of both male and female worms within 72 h of incubation (Figure 3A). FB9 showed mortality effects only on the last day of incubation (192 h of contact) and only at concentrations of 0.338, 0.405 and 0.473 mM (Figure 3C).

**Table 2.** Mating (M) and egg laying (EL) of adult worms of *S. mansoni* after contact with eugenol derivatives FB1, FB4, and FB9 using ED_50_ concentration, besides praziquantel (PZQ) at 0.0064 mM.

Compound	Evaluation time (hour)					
	0-24		48-72		96-192	
	M (%)	EL (%)	M (%)	EL (%)	M (%)	EL (%)
**FB1**	25.0	0.0	25.0	0.0	25.0	0.0
**FB4**	25.0	0.0	25.0	0.0	25.0	0.0
**FB9**	50.0	0.0	50.0	0.0	50.0	0.0
**PZQ**	100.0	0.0	100.0	0.0	100.0	0.0
**RPMI-1640**	100.0	40.0	100.0	60.0	100.0	100.0
**Methanol**	100.0	40.0	100.0	60.0	100.0	100.0

RPMI-1640: culture medium.

### Use of fluorescent probes for *in vitro* evaluation of the effects of eugenol on *S. mansoni* adult worms

**Figure 4.**
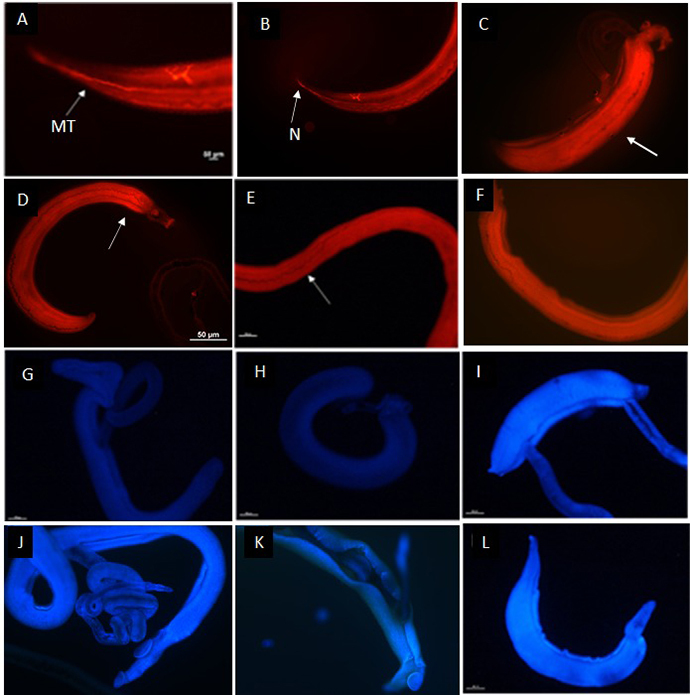
(A-F) Resorufin marking showing damage to the excretory system of *Schistosoma mansoni* adult worms by eugenol derivatives. (G-L) In addition, the marking of tegument lesions in *S. mansoni* adult worms by the Hoechst 33258 probe is shown. (A) Adult male worm exposed to RPMI-1640 (negative control) with fluorescent labelling, indicating a normally functioning excretory system. Arrow: main tubule (MT). (B) An adult female worm incubated with methanol diluent, indicating a normally functioning excretory system, nephridiopore (N), and ramifications of the excretory system. (C) Couple of worms exposed to PZQ (pharmacological control). (D) Male exposed to 0.312 mM of FB1. (E) Female exposed to 0.161 mM of FB4. (F) Male exposed to 0.338 mM of FB9. (C, D, E, F) Fluorescent labelling diffused throughout the body of the parasite, indicating that the excretory system is not working due to the action of the substances (as shown in the arrows). (G) A pair of worms incubated with RPMI-1640 culture medium. (H) A pair of worms incubated with methanol diluent. (I) A pair of worms exposed to PZQ worms showing fluorescent labelling throughout. (J) Adult male worm exposed to 0.312 mM of FB1. (K) A pair of worms exposed to 0.161 mM of FB4. (L) A male worm exposed to 0.338 mM of FB9. (I, J, K, L) The fluorescent areas indicate intense lesions.

*In vitro* evaluation of the excretory system activity in adult worms was also performed by using resorufin incubated with eugenol derivatives (FB1, FB4 and FB9), besides control groups (RPMI-1640 and Methanol) and PZQ. Figure 4A and 4B shows the functioning excretory system of adult *S. mansoni* worms in the culture medium and methanol groups after labeling with the resorufin probe. Notably, the fluorescence is delimited along the parasite length as it expels the probe, indicating a normally functioning excretory system. Besides, it was possible to notice the main tubule and nephridiopore in Figures 4A and 4B, respectively. The parasites exposed to the PZQ (0.0064 mM) pharmacological control (Figure 4C) showed a differentiated fluorescence pattern, which emissions scattered throughout the body, indicating an inactive excretory system. Parasites exposed to 0.312 mM FB1, 0.161 mM FB4 and 0.338 mM FB9 (Figures 4D, 4E and 4F) also displayed a damaged excretory system because these groups showed a similar probe distribution to that of the PZQ-treated worms.

Integument damage was assessed using the Hoechst 33258 probe (Figure 4G-4L). The absence of any fluorescent marking in the controls (culture medium and methanol alone) confirmed that the membrane integrity was intact (Figures 4G and 4H). By contrast, exposure to the eugenol derivatives resulted in clear injury, as indicated by the intense fluorescence emitted by the probe in the damaged regions (Figure 4I, 4J, 4K and 4L). Figures 4I, 4J, 4K and 4L show parasites exposed to PZQ (0.0064 mM), FB1 (0.312 mM), FB4 (0.161 mM), and FB9 (0.338 mM), respectively.

### Scanning electron microscopy evaluation of eugenol derivative-induced integumentary damage

**Figure 5.**
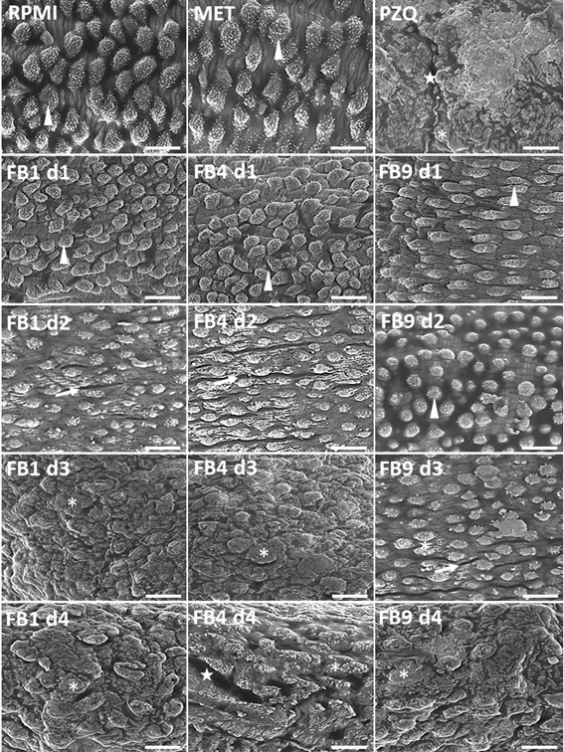
Scanning electronic photomicrographs of the integument of *Schistosoma mansoni* adult worms untreated and treated with different doses of eugenol derivatives FB1, FB4, and FB9. Controls: RPMI-1640 supplemented medium, RPMI-1640 medium plus 25.0 µL of methanol - MET (the vehicle used as solvent for the samples) and with PZQ (0.0064 mM pharmacological control). Representative images from the end of a 192-hour observation period. RPMI-1640 and MET: integument with preserved morphology, showing prominent tubercles and well-defined spicules and PZQ: tubercle collapse, spicule disappearance and integumentary contraction bands. Arrowhead: tubercles with spikes. Arrow: contraction bands. Asterisk: collapsing tubercles in degeneration. Star: erosion of the integument. FB1 and FB4, complete loss of the integument grooves at dose 3 (d3). *d1 and d2: doses below ED_50_ to FB1, 0.104 and 0.208 mM; FB4, 0.080 and 0.161 mM; FB9, 0.203 and 0.270 mM. *d3 and d4: doses above ED_50_ to FB1, 0.416 and 0.521 mM; FB4, 0.242 and 0.322 mM; FB9, 0.405 and 0.473 mM.

As indicated in Figure 5, no microstructural changes were observed in the grooves, tubercles, and spicules of the integument in parasites treated with FB1, FB4, or FB9; which ED_50_ was 0.324, 0.167, and 0.380 mM, respectively. Treatment with doses just below (d1 and d2) the ED_50_ (FB1, 0.104 and 0.208 mM; FB4, 0.080 and 0.161, besides FB9, 0.203 and 0.270 mM) caused a reduction or disappearance of spicules, a flattening of the tubercles, and the formation of integumentary contraction bands. At doses above (d3 and d4) the ED_50_ (FB1, 0.416 and 0.521 mM; FB4, 0.242 and 0.322 mM, besides FB9, 0.405 and 0.473 mM) the treatments induced marked integumentary damage, as evidenced by the complete collapse of the tubercles and the disappearance of the spicules and integumentary grooves. In addition, integumentary erosion was also identified in worms exposed to a high dose of FB4 (0.322 mM). No differences were detected in the images of the control group in relation to the groups treated with the lower doses of the eugenol derivatives (data not shown).

### Comparison of the *in vitro* schistosomicidal activity of eugenol derivatives *versus* verapamil or ouabain against *S. mansoni* adult worms

The mechanism of action of eugenol derivatives against adult *S. mansoni* worms was investigated by treating with a combination of verapamil and either FB1, FB4, or FB9 (at their ED_100_ concentrations for 100% mortality), followed by morphological evaluation. FB1 and FB4 alone or in combination with verapamil caused the death of 100% of the worms in the first hours of contact, suggesting that these two eugenol compounds do not interact with calcium channels. FB9 caused 50% mortality within 2 h of contact but did not cause mortality after 2 h of contact when combined with verapamil (Figure 3D). Observations of active motility, mating, and oviposition were also possible at this time and indicated some dependence on calcium channels for the action of FB9. FB9, in combination with verapamil, induced 100% mortality after 24 h. Thus, evaluation of the worms over the seven days of the experiment confirmed 100% worm mortality (Figure 3D). 

The worms showed moderate motility when treated the eugenol derivatives in combination with ouabain, indicating some relationship between the eugenol derivatives and the Na^+^/K^+^-ATPase. However, after 72, 24, and 192 h of incubation, FB1, FB4, and FB9, respectively, caused 100% mortality. Treatment with FB1 and FB4 alone resulted in 100% mortality in the first 2 h of contact, while FB9 alone caused a 50% mortality in the first two hours (Figure 3E).

The control groups treated with PZQ and culture medium showed no differences in mortality with or without ouabain, except after 192 h, when 100% mortality was observed for the combination with ouabain in both treatments (Figure 3E). 

### *In silico* analysis

Molecular docking was performed to evaluate the interactions between eugenol derivatives and a homology model of the *S. mansoni* Na^+^/K^+^-ATPase. Table 3 lists the predicted binding energies for the most stable ligand-enzyme complexes and the main interactions in the same region where ouabain interacts. 

**Figure 6.**
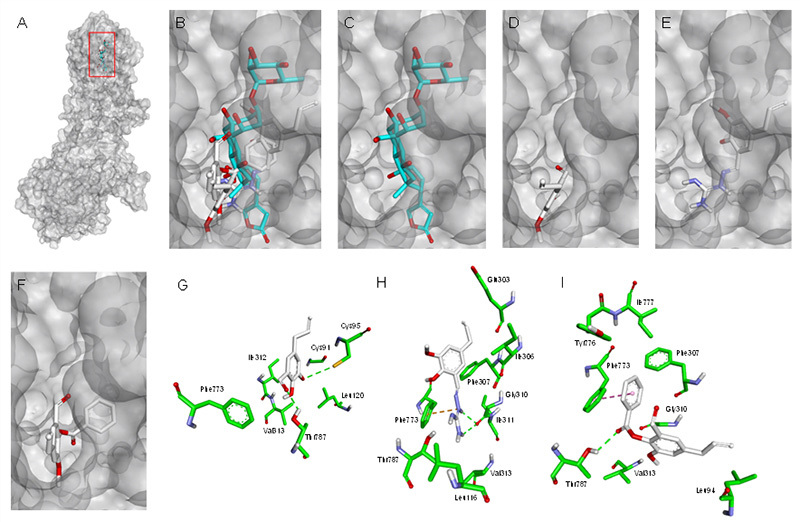
Docking poses and interactions of eugenol derivatives in the *Schistosoma mansoni* Na+/K+-ATPase alpha subunit. **(A)** Ligand interaction region, highlighted by the red square, in the *S. mansoni* Na+/K+-ATPase alpha subunit model (grey surface representation). **(B)** Relative position of docking poses of eugenol derivatives (white carbons). A modelled ouabain (cyan carbons) was included as reference. **(C, D, E, F)** Individual poses assumed by ouabain, FB1, FB4, and FB9, respectively, in the *S. mansoni* Na+/K+-ATPase alpha subunit model. **(G, H, I)** Interactions of FB1, FB4, and FB9 (white carbons), respectively, in the ouabain active site of the *S. mansoni* Na+/K+-ATPase alpha subunit (green carbons). Cation-pi interaction (dotted yellow line), hydrogen bonds (dotted green lines) and pi-pi stacking interactions (dotted pink line) are indicated.

Figure 6A-6F shows how the eugenol derivatives tend to occupy regions explored by the lactone ring and the steroid core of ouabain, in accordance with the crystallographic model (PDB ID 3A3Y) presented by Ogawa et al. [22]. The complexes between the homology model and the eugenol derivatives are formed by polar and hydrophobic interactions, as shown in Table 3, especially in relation to the residue Phe773. While FB1 can interact by Van der Waals forces with Phe773 (Figure 6G), the nitrogenous side chain of FB4 performs cation-pi interactions (Figure 6H), whereas the aromatic side chain of FB9 interacts by T-shaped pi-pi stacking (Figure 6I).

Both FB1 and FB9 maintain a hydrogen bond between the hydroxyl bound to C14 and the 

Thr787 residue (Figure 6G and 6I). The nitrogenous side chain of FB4 is the closest group to this residue; however, the conformation assumed by this molecule favors hydrogen bonds with Gly310 rather than with Thr787 (Figure 6H).

**Table 3.** Docking results for eugenol derivatives FB1, FB4, and FB9 to the channel of the *S. mansoni* Na+/K+-ATPase.

Ligand-enzyme interaction					
Compound	Binding energy (kcal/mol)	Van der Waals	Hydrogen bond	Cation-pi	pi-pi stacking
**FB1**	-5.0	Gly91, Leu120, Gly310, Ile312, Val313, Phe773	C=O:Thr787; H_3_CO:Cys95	-	-
**FB4**	-6.1	Leu116, Glu303, Ile306, Phe307, Ile311, Val313, Thr787	(H_2_N)_2_C=N-N=C:Gly310	(H_2_N)_2_C=N-N=C:Phe773	-
**FB9**	-6.3	Leu94, Phe307, Gly310, Val313, Tyr776, Ile777	Ar’-COO-Ar:Thr787	-	Ar’-COO-Ar:Phe773

## Discussion

Natural products like eugenol represent an endless source of opportunities for the development of new compounds with unique structural patterns. Eugenol is a natural phenylpropanoid found in the essential oils of cloves, cinnamon, and sassafras [25]. 

A recent study by El-kady et al. [12] described the antischistosomal effect of eugenol on *S. mansoni* infection in a mouse model. They reported that the eugenol-treated group exhibited a reduction in egg density in the intestinal walls and a significant reduction in total worm burden [12]. *In vitro* studies have shown that eugenol derivatives also have antiparasitic activities ranging from antitrypanosomal [7], antileishmanial [9], and antigiardial [26] to antimalarial [8] and antischistosomal activities [12]. According to Glaser et al. [11], simple modifications of the eugenol scaffold can improve the vacuole formation and consequent schistosome death observed with eugenol [11]. The eugenol derivatives synthesized here exhibited schistosomicidal effects, with some activity at concentrations lower than 0.521, 0.322 and 0.473 mM for FB1, FB4, and FB9 (Figure 3), respectively. The FB4 compound showed the best ED_50_ at 0.167 mM (p < 0.05, Table 1).

The qualitative analysis of *S. mansoni* activity is the usual method for evaluating drug performance. In this sense, the neuromotor system of *S. mansoni* constitutes an interesting target for studying new schistosomicidal compounds. The preliminary studies by Bennett and Bueding [27] to evaluate the motor activity of *S. mansoni* on a visual scale show the pioneering strategy of this type of analysis. Therefore, in the present work, the motor activity of the worm was also used as a qualitative parameter for evaluation, with a movement scale of 0-3 established to assess the motility of parasites in contact with different concentrations of eugenol derivatives [28]. According to the findings of this study, the reduction in motor activity was directly proportional to the concentration and the incubation period of the evaluated compounds (Figure 2). Once again, the eugenol derivative FB4 caused the greatest reduction in motor activity in relation to control groups (RPMI-1640 and methanol), even at a concentration of 0.121 mM.

The *S. mansoni* parasite differs from other hermaphroditic trematodes in that it has a marked sexual dimorphism, with co-dependency observed between males and females. This fact occurs because, in the absence of the male worm, there is no possibility of perpetuation of the female's sexual development and maturation [28]. Furthermore, the gynecophore channel present in the male is responsible for part of the female's development and is also the structure where mating and subsequent sexual maturation occur, together with egg production [29]. In the present work, 75.0, 75.0 and 50.0% of the worm couples exposed (at ED_50_ concentration) to FB1, FB4 and FB9, respectively separated after 192 h of contact. Besides, no oviposition was observed at any time during the experiment even in the situation of mating. According to Pereira et al. [30] aryl-thiazole derivatives can down regulate genes involved in egg biosynthesis, besides in some situation the combination of these compounds can affect the oviposition although they were not effective in enhancing couples unpairing.

A previous study [31] reported the observation of physiological and morphological changes, apart from the lethal effect on the studied organism, by *in vitro* screening of chemical substances. Our findings revealed that all three eugenol derivatives caused mortality of the worms in a dose- and time-dependent manner. In other words, the lethality of the parasites was directly dependent on the assessed concentration and the exposure period (Figures 3A, 3B, and 3C).

The excretory system activity in adult worms treated with FB1, FB4, and FB9 was assessed with the resorufin probe, as described by Castro et al. [19]. All three eugenol derivatives interfered with the activity of the excretory system, probably by inhibition of P-glycoprotein (Pgp). According to Sato [17], the resorufin probe is a fluorescent salt that works as a substrate of the Pgp modulator, which is a protein expressed in the excretory epithelium of *S. mansoni* adult worms. Figure 4A-4F shows that FB1, FB4 and FB9 caused damage to the excretory system of the adult worms when applied at 0.312, 0.161 mM and 0.338, respectively. The excretory system of schistosomes seems to play an important role in the host-parasite interaction and can be used as target of signalling molecules such as phosphatases and proteases [32]. Besides, the evaluation of excretory activity may represent a method to identify resistant (or less susceptible) isolates of the *Schistosoma* because PgP or homologous proteins have an important role in the elimination of various drugs [33]. Therefore, Pgp and multidrug resistance-associated proteins substrates on the excretory system can be a potentially attractive target for new antischistosomals [34].

Integument damage in adult worms exposed to eugenol derivatives was evaluated *in vitro* with the Hoechst 33258 probe, as described by Castro et al. [19]. This probe emits fluorescence when it binds to cellular DNA. The results corroborated the integumentary lesions observed in the previous inverted microscopy analyses, where bubbles indicated injury to the integument. The integument of *S. mansoni* adult worms has an important function in host-parasite interactions, making it a key target in the search for new schistosomicidal agents. The integument also performs an essential role in evasion of the immune response in the human body [35]. Figure 4G-4L shows fluorescent staining, indicating that these substances caused damage to the integument of the adult *S. mansoni* worms.

Dias et al. [20] reported that scanning electron microscopy (SEM) is a valuable tool to evaluate integumentary lesions in *S. mansoni* adult worms, which cannot be resolved by bright field microscopy. Thus, SEM revealed marked morphological evidence drug toxicity, such as flattening or disappearance of spicules and tubercles and the presence of bubbles, desquamation, erosion, and contraction bands in *S. mansoni* integument. The images in Figure 5 show that all three eugenol derivatives had a dose-dependent toxic effect on the integument of the adult *S. mansoni* worms. Once again, FB4 caused more severe integumentary damage (especially erosions) compared to the other molecules investigated.

Marchese et al. [36] investigated the mechanism of action of eugenol in relation to different pathogenic microorganisms, such as bacteria, to explain the biological activity of this compound. A rupture of the cytoplasmic membrane was observed that, in turn, increased the non-specific permeability of the membrane and affected the transport of ions and ATP [37]. Another study reported a mechanism of action related to the modification of the fatty acid profile of the bacterial membrane [38]. Hyldgaard et al. [39] demonstrated that the capacity of eugenol to cause cell cytotoxicity was due to the production of reactive oxygen species (ROS) that consequently led to the inhibition of cell growth, disruption of the cell membrane, and DNA damage, ultimately resulting in cell decomposition and death. These authors also observed that eugenol was active against some bacterial enzymes, such as proteases, histidine carboxylase, amylase, and ATPase.

Shang et al. [40] evaluated the possible mechanism of action of eugenol derivatives against *Psoroptes cuniculi* by determining the inhibition of acetylcholinesterase (AChE) and glutathione-S-transferase (GST), and cytochrome P450 (P450). These authors also demonstrated that eugenol significantly inhibited enzymatic activity against mites. Bennis et al. [41] observed morphological changes in the envelopes of the fungi *S. cerevisiae* due to eugenol, as did Braga later in *C. albicans* [42]. The proposed mechanism of action of eugenol against fungi was related to its chemical characteristics. Eugenol is a lipophilic substance, so its activity could involve its penetration into the fatty acyclic chains of the lipid bilayer of the membrane and disruption of membrane fluidity and permeability. Ahmad et al. [43] verified the inhibition of the H^+^-ATPase activity of *Candida* spp. by eugenol, in addition to inhibition of the H^+^ excretion stimulated by glucose.

Noël et al. [44] determined that PZQ has a toxic effect on the adult worms *in vitro* through its action on calcium channels, and that PZQ toxicity could be canceled in the absence of external calcium or in the presence of verapamil, a calcium antagonist. This mechanism was attributed to the modulation of the β subunit of the L-type calcium channel, which in turn leads to its opening [45]. In the present work, adult worms in contact with PZQ alone for only 2 h presented the predicted alterations in morphology (Figure 4D), characterized by static paralysis, integumentary rupture, and ultimately worm death [46]. However, verapamil inhibited PZQ activity, as indicated by retention of suction cup movements and some motility after 72 h of contact.

The perpetuation (transmission) and pathology of schistosomiasis is known to require the production of eggs by adult female worms. Biochemically, this production involves the biosynthesis and storage of eggshell proteins in vitelline cells, exocytosis of the eggshell proteins from these cells, and crosslinking of the eggshell proteins by the activity of the phenol oxidase enzyme. Although these events are not fully understood, some evidence suggests that calcium has a role in regulating schistosome reproduction [47]. In this sense, the eugenol derivative FB9 may be acting on the same calcium channel affected by verapamil, since the combination of FB9 and verapamil did not reduce motility and/or cause greater mortality of the worms in the first hours of contact (this mortality occurred only in the presence of FB9).

Ouabain has been used as a standard inhibitor of Na^+^/K^+^-ATPases, so it was used to assess the involvement of the Na^+^/K^+^-ATPase in the mechanism of action of the eugenol derivatives. The eugenol derivatives were used at their ED_100_ concentrations to ensure 100% mortality. Usta et al. [48] observed that eugenol from cloves and cinnamon inhibited the Na^+^/K^+^-ATPase activity of rat liver, indicating that Na^+^/K^+^-ATPases may be possible targets of eugenol derivatives. Disruption of Na^+^/K^+^-ATPase activity can result in several biological responses, such as electrolyte imbalances and breakdowns in mitochondrial function. Figure 4E shows a time-dependent relationship of eugenol derivatives and Na^+^/K^+^-ATPase activity, as a delay in the motility/death of the parasites was observed upon exposure to ouabain. Nevertheless, inhibition of Na^+^/K^+^-ATPase did not completely inhibit the activity of eugenol, thereby demonstrating that these compounds, and FB4 in particular (this compound did not show a time-dependent relationship), may act on targets other than the parasite Na^+^/K^+^-ATPase.

The main time- and concentration-dependent effects of the eugenol derivatives were the bubbling and shedding of the damaged integument and the cessation of digestive system activity, along with a reduction in motility, the absence of mating and consequent oviposition, and increased mortality. These compounds most likely alter the homeostasis of the Na^+^/K^+^-ATPase, thereby contributing to the dysregulation of the maintenance membrane potential of the worm's nerve and muscle cells. However, one noteworthy point is that mating, and oviposition were similar with or without ouabain, in agreement with the findings with verapamil. Mortality was caused by FB1, FB4, and FB9 after 72, 24 and 168 h, respectively, when supplied alone and after 192, 24, and 192 h, respectively, when combined with ouabain. Notably, the response to FB4 was the same in the presence or absence of ouabain.

Comparison of the docking poses of eugenol derivatives with that assumed by ouabain in a reference crystallographic model (PDB ID 3A3Y) [22], revealed that the ligands tend to occupy regions explored by the lactone ring and the steroid core of ouabain (Figure 6A-6F). The anchoring of the ouabain steroid core is facilitated by contact with hydrophobic residues in the enzyme channel. Among these residues, Phe773 appears to play an important role in interacting with this group [22]. All the eugenol derivatives maintained interactions with this residue, but in different ways. Another critical point in the interaction of the ouabain steroid core with Na^+^/K^+^-ATPase involves a hydrogen bond between the hydroxyl of C14 and Thr787 [22]. Although FB1 and FB9 maintained the same type of interaction with this residue via the carbonyl oxygen, the nitrogenous side chain of FB4 interacts with Gly310 (Figure 6G-6I).

The failure to undergo this interaction may reflect a compromise in the permanence of this ligand at the active site of the enzyme so that it cannot exert an antiparasitic effect through this mechanism. The combined findings of the *in silico* and biochemical analysis indicate that FB4 cannot form the same crucial interaction as FB1 and FB9 with the Na^+^/K^+^-ATPase to inhibit the enzyme function.

## Conclusion

The FB1 and FB9 interacted best with the Na^+^/K^+^-ATPase, as observed by both *in silico* and biochemical analysis. Interestingly, they were the derivatives that had the highest ED_50_ but the least effect on the integument of the adult worms. Conversely, the *in silico* studies indicated that FB4 would not form a crucial interaction with the Na^+^/K^+^-ATPase to inhibit the pump function, in agreement with its biochemical activity. Surprisingly, FB4 had the lowest ED_50_, ED_90_ and ED_100_ (p < 0.05) and the greatest effect on the integument of the parasite, as observed by the fluorescent marker probes and scanning electron microscopy investigations. Therefore, the eugenol derivatives appear to act more effectively when they work on the integument of the adult *S. mansoni* worms.
